# Appendicitis in pregnancy, higher rate of perforation compared to nonpregnant patients

**DOI:** 10.1515/crpm-2024-0042

**Published:** 2025-05-05

**Authors:** Mary Beth Janicki, Reinaldo Figueroa, Dorothy Wakefield, Jennifer Hill, David Shapiro

**Affiliations:** Division of Maternal Fetal Medicine, Department of Obstetrics and Gynecology, Trinity Health of New England, Saint Francis Hospital and Medical Center, Hartford, CT, USA; Frank H. Netter MD School of Medicine at Quinnipiac University, North Haven, CT, USA; UConn School of Medicine, Farmington, CT, USA; UCONN Center on Aging, UConn Health, Farmington, CT, USA; Division of Maternal Fetal Medicine, Department of Obstetrics and Gynecology, Hartford Hospital, Hartford, CT, USA; Department of Surgery, Saint Francis Hospital and Medical Center, Hartford, CT, USA

**Keywords:** appendicitis, pregnancy, diagnosis, appendectomy

## Abstract

**Objectives:**

To compare clinical presentation and diagnostic evaluation to identify differences in treatment between pregnant and nonpregnant patients with appendicitis.

**Methods:**

Retrospective case-control study comparing 12 pregnant and 60 nonpregnant, age-matched patients who had an appendectomy for acute appendicitis (pathology confirmed) between January 1, 2011 and June 30, 2019. We compared maternal characteristics, laboratory test results, physical examination findings, diagnostic work-up, surgical modality, and surgical outcomes.

**Results:**

There was no difference in symptom profile and pain intensity at presentation between groups. More pregnant patients had right upper quadrant tenderness (83.3% vs. 31 %, p=0.03) and were more likely to have more than one imaging diagnostic modality (75% vs. 15 %, p<0.01). In nonpregnant patients, computed tomography was the main diagnostic modality (90 %) whereas there was more variation in imaging for pregnant patients. For pregnant patients, time from presentation to surgery (20.0 ± 11.8 h vs. 9.9 ± 4.9 h; p=0.01) and time from presentation to receipt of antibiotics (14.5 ± 12.0 h vs. 5.9 ± 3.2 h, p<0.01) were twice that of nonpregnant patients. Surgery duration was similar between groups (pregnant: 54.8 ± 31.3 min vs. nonpregnant: 45.6 ± 19.5 min, p=0.34). All nonpregnant patients underwent laparoscopic appendectomy. Seven pregnant patients underwent laparoscopy, three had laparotomy, and two began with laparoscopy that was converted to laparotomy. More pregnant patients perforated (25 % vs. 3.3 %, p=0.03).

**Conclusions:**

Despite having similar presentations, it took twice as long to treat pregnant patients with antibiotics and perform an appendectomy. More perforations occurred in pregnant patients compared to nonpregnant patients.

## Introduction

Pregnant patients presenting with acute abdominal pain can create a diagnostic challenge. The physiologic changes of pregnancy, gastrointestinal discomfort, displacement of bowel by the enlarged uterus (elevating the appendix), and the physiologic leukocytosis of pregnancy, can obscure the clinical presentation of appendicitis and may be associated with delayed diagnosis and management [1]. Gastrointestinal conditions that require non-obstetric surgery during pregnancy include appendicitis, cholecystitis, pancreatitis, intestinal obstruction, intestinal ischemia, and perforation. Appendicitis is the most common, occurring in 1/500 to 1/1,700 pregnancies, with the incidence unchanged by pregnancy [1], [2], [3]. Delay in diagnosis can lead to rupture, peritonitis, sepsis, pregnancy loss, and preterm birth.

Diagnosis of appendicitis in nonpregnant patients often relies on contrast-enhanced computed tomography (CT) imaging which has decreased the negative appendectomy rate (rate of removal of a normal appendix) from 23 %, relying on clinical diagnosis, to 1.7 % [4]. Use of CT in pregnancy is discouraged because of fetal exposure to ionizing radiation [5]. Ultrasound (US) has been proposed as the initial imaging modality in pregnancy because it is repeatable, noninvasive, inexpensive, and does not use ionizing radiation [6]. US is operator dependent and can be difficult to interpret particularly with obesity or a retrocecal appendix. Magnetic resonance imaging (MRI) is proposed as a secondary test, when ultrasound is inconclusive, and has a high sensitivity, specificity, and positive and negative predictive values [7], 8].

The perception is that pregnant patients with appendicitis present with signs, symptoms, and laboratory values that differ from nonpregnant patients, for instance, a change in location of pain. This perception, combined with reliance on CT for diagnosis in the nonpregnant population, could result in delay in diagnosis and treatment among pregnant patients. The objective of this study is to compare clinical presentation and diagnostic evaluation to identify any differences in treatment and rate of perforation between pregnant and nonpregnant patients.

## Materials and methods

This study was approved by the Institutional Review Board (SFH-19-47). We conducted a retrospective case-control study comparing pregnant and nonpregnant patients who had an appendectomy for the preoperative diagnosis of acute appendicitis and diagnosis confirmed on pathology. Female patients between 18 and 50 years old who underwent appendectomy between January 1, 2011, and June 30, 2019, were identified in our electronic medical record system Epic (Verona, WI). Pathology reports were reviewed to identify patients with the diagnosis of appendicitis. Acute appendicitis was characterized as variable acute inflammation with predominance of neutrophils involving the appendiceal wall. Chronic appendicitis was characterized as chronic inflammation, fibrosis, granulomatous reaction, and infiltration of lymphocytes, eosinophils, and plasma cells. Among these patients, cases were defined as patients with pregnancy confirmed by ultrasound or positive urine human chorionic gonadotropin-pregnancy test. Five nonpregnant control patients were matched temporally and by proximity in age to each pregnant case.

We collected demographic information: age, ethnicity, and body mass index (BMI). Chief complaint (presence of pain and location), number of days of symptoms, pain score, and associated symptoms, were extracted. Vital signs, physical examination (abdominal tenderness and location), type of physician performing the initial assessment (emergency medicine physician, obstetrician, or surgeon), laboratory test results (white blood cell count (WBC), hemoglobin, hematocrit, aspartate aminotransferase (AST), alanine aminotransferase (ALT), amylase, and lipase), and diagnostic work up (CT, MRI, US) were obtained. We noted surgical modality (laparoscopy, laparoscopy converted to laparotomy, or laparotomy), timing of interventions (time from presentation to diagnosis, time from presentation to treatment), duration of surgery, and duration of hospital stay (postoperative stay, total hospital stay). Postoperative complications and pregnancy outcomes when available were recorded. Pathology reports were reviewed to confirm the diagnosis of appendicitis and perforation status.

We compared maternal characteristics, laboratory test results, physical examination findings, diagnostic work-up, surgical modality, and post-surgical outcomes between groups. Descriptive statistics, mean ± standard deviation and frequencies (%), were calculated as appropriate for demographics. Chi-square analyses or Fisher’s Exact test (when cell size <5) were used to compare categorical variables and T-tests were used to compare continuous variables. Postoperative stay and total hospital length of stay were also reported as medians and interquartile ranges (IQR) using the Wilcoxon Rank Sum test because of skewed distributions. Statistical analysis was performed with the SAS version 9.4 software (SAS Institute, Inc., Cary, NC). All tests used a 2-sided alpha level of significance of 0.05.

## Results

Five hundred 70 female patients underwent appendectomy during the study period. 12 pregnant patients were compared to 60 nonpregnant controls. Mean gestational age at presentation was 19.8 ± 9.4 weeks. There were no significant differences in age, ethnicity, and BMI between groups (Table 1). There was no difference in symptom profile at presentation between pregnant and nonpregnant patients (Table 1). Ninety-two percent of patients in both groups reported right lower quadrant pain or generalized abdominal pain. The intensity of pain was similar for both groups. Associated symptoms (nausea, vomiting, diarrhea, anorexia) were seen with similar frequency (Table 1).

**Table 1:** Demographic characteristics and presenting symptoms of the study population.

	Pregnant n=12	Non-pregnant n=60	p-Value
Age, years	28.7 ± 6.1	28.8 ± 5.9	0.94
Ethnicity			0.46
White	50.0 (6)	50.0 (30)	
Black	8.3 (1)	18.3 (11)	
Hispanic	25.0 (3)	26.7 (16)	
Asian	16.7 (2)	5.0 (3)	
BMI, kg/m^2^	29.8 ± 5.0 (n=11)	28.7 ± 8.4 (n=57)	0.68
Chief complaint – pain			0.48
Right lower quadrant	50.0 (6)	46.7 (28)	
Right-sided abdominal	0 (0)	6.6 (4)	
Generalized abdominal	41.7 (5)	45.0 (27)	
Epigastric	8.3 (1)	1.7 (1)	
Days of symptoms			0.27
1 day	50.0 (6)	66.7 (40)	
>1 day	50.0 (6)	33.3 (20)	
Pain score on presentation			0.86
3 to 5	25.0 (3)	28.3 (17)	
6 to 8	33.3 (4)	38.3 (23)	
9 to 10	41.7 (5)	33.3 (20)	
Other symptoms			
Nausea	83.3 (10)	80.0 (48)	1.0
Vomiting	50.0 (6)	41.7 (25)	0.59
Diarrhea	16.7 (2)	21.7 (13)	1.0
Anorexia	50.0 (6)	61.7 (37)	0.37
Pain radiates	41.7 (5)	21.7 (13)	0.14

Data presented as % (n) and mean + SD, where applicable.

Table 2 shows the vital signs, laboratory findings and physical examination of the study population. Vital signs, obtained before any analgesia or acetaminophen were given, were similar among both groups. Hemoglobin (12.0 ± 1.5 g/dL vs. 13.1 ± 1.2 g/dL, p<0.01) and hematocrit (36.2 ± 4.2 % vs. 39.7 ± 3.1 %, p<0.01) were lower for pregnant patients. There was no difference in the other laboratory values (WBC, AST, ALT, amylase, lipase) on presentation (Table 2). The clinical diagnosis of appendicitis was made 95 % of the time for nonpregnant patients by an emergency medicine physician compared to 50 % of the time for pregnant patients (p<0.01). More pregnant patients had right upper quadrant tenderness than nonpregnant patients (83.3% vs. 31 %, p=0.03). Otherwise, there was no difference in tenderness on abdominal exam (Table 2).

**Table 2:** Vital signs, laboratory findings and physical examination of the study population.

	Pregnant (n=12)	Non-pregnant (n=60)	p-Value
Vital signs			
Heart rate	99.2 ± 15.0	89.4 ± 16.5	0.06
Systolic BP, mmHg	124.8 ± 19.2	123.2 ± 13.7	0.7
Diastolic BP, mmHg	73.2 ± 12.2	76.8 ± 10.9	0.30
Maximum temperature, °F	99.7 ± 1.7	99.0 ± 0.9	0.17
Temperature >100.5 °F	16.7 (2)	8.3 (5)	0.30
Laboratory values			
WBC count, × 1,000/µL	13.0 ± 2.5	12.0 ± 3.9	0.43
Neutrophils, %	80.8 ± 8.8 (n=10)	77.2 ± 10.7 (n=57)	0.32
Hemoglobin, g/dL	12.0 ± 1.5	13.1 ± 1.2	<0.01
Hematocrit, %	36.2 ± 4.2	39.7 ± 3.1	<0.01
AST, U/L	16.4 ± 6.9 (n=12)	17.7 ± 6.8 (n=46)	0.56
ALT, U/L	12.6 ± 7.0 (n=12)	12.5 ± 6.4 (n=46)	0.99
Amylase, U/L	31.0 ± 13.3 (n=5)	40.6 ± 15.0 (n=28)	0.19
Lipase, U/L	26.5 ± 37.3 (n=8)	15.0 ± 10.3 (n=31)	0.42
1st examiner			<0.01
Emergency medicine	50.0 (6)	95.0 (57)	
Obstetrician	50.0 (6)	0 (0)	
Surgeon	0 (0)	1.7 (1)	
Diagnosed outpatient	0 (0)	3.3 (2)	
Physical exam			
RLQ tenderness	81.8 (9) (n=11)	94.4 (51) (n=54)	0.20
RUQ tenderness	83.3 (5) (n=6)	31.0 (9) (n=29)	0.03
LUQ tenderness	50.0 (3) (n=6)	15.4 (4) (n=26)	0.10
LLQ tenderness	42.9 (3) (n=7)	41.4 (12) (n=29)	1.0
Rebound tenderness	40.0 (4) (n=10)	26.8 (11) (n=41)	0.45
Guarding	33.3 (3) (n=9)	46.8 (22) (n=47)	0.72
Epigastric tenderness	60.0 (3) (n=5)	17.4 (4) (n=23)	0.08
Diffuse tenderness	50.0 (4) (n=8)	16.7 (4) (n=24)	0.15
Abdominal distension	0 (0) (n=6)	8.1 (3) (n=37)	1.0

Data presented as % (n) and mean + SD, where applicable. BP, blood pressure; WBC, white blood cell; AST, aspartate aminotransferase; ALT, alanine aminotransferase; RLQ, right lower quadrant; RUQ, right upper quadrant; LUQ, left upper quadrant; LLQ, left lower quadrant.

Pregnant patients were more likely to have more than one imaging modality (75 % vs. 15 %, p=<0.01). An ultrasound was done first in patients having more than one imaging modality. In nonpregnant patients CT was the diagnostic modality (90 %) whereas there was more variation in imaging for pregnant patients. Two pregnant patients had a CT, four had MRI, four had US, and two were diagnosed clinically (Table 3). For pregnant patients, the time from presentation to surgery was twice that of nonpregnant patients (20.0 ± 11.8 h vs. 9.9 ± 4.9 h, p=0.01). There was no difference in time from presentation to surgery among patients <20 weeks’ pregnant (n=6) compared to patients >20 weeks’ pregnant (n=6) [mean: 17.1 ± 8.5 h vs. 23 ± 14.6 h; median (IQR): 14.2 (13.3, 23.9) h vs. 21.8 (11, 36.9) h, p=0.41]. The time from presentation to receipt of antibiotics was more than twice that of nonpregnant patients (14.5 ± 12.0 h vs. 5.9 ± 3.2 h, p<0.01). Three nonpregnant patients received antibiotics before imaging. For pregnant patients the time from presentation to first imaging study (8.0 ± 11.1 h vs. 3.2 ± 2.0 h, p=0.16) and time from initial imaging study to operating room (12.0 ± 8.9 h vs. 6.7 ± 4.6 h, p=0.66) were twice that of nonpregnant patient; however, the differences were not statistically significant. Duration of surgery was similar between groups even with pregnant patients having laparotomies or conversion to laparotomy (pregnant: 54.8 ± 31.3 min vs. nonpregnant: 45.6 ± 19.5 min, p=0.34) (Table 3). All nonpregnant patients underwent laparoscopy. Of the 12 pregnant patients, seven underwent laparoscopy, three had laparotomy, and two began with laparoscopy that was converted to laparotomy because of technical difficulties. None of the 72 patients were initially managed medically; once the diagnosis was made, they underwent surgery. There were no postoperative complications. Pregnant patients had longer postoperative stays [mean: 51.1 ± 34.9 h vs. 18.6 ± 14.3 h, p<0.01; median (IQR): 56.1 (22.2, 63.3) h vs. 16.3 (9.6, 21.1) h, p<0.01] and longer hospitalizations [mean: 65.9 ± 39.1 h vs. 28.8 ± 15.9 h, p<0.01; median (IQR): 67.2 (30.6, 84.7) h vs. 25 (18.4, 33.4) h, p<0.01]. All patients had acute appendicitis on pathology; however, more pregnant patients had perforation confirmed on pathology compared to nonpregnant patients (25 % vs. 3.3 %, p=0.03). Three nonpregnant patients with chronic appendicitis had acute infection superimposed on chronic inflammation on pathology (Table 3).

**Table 3:** Diagnostic studies, surgical interventions, timing of interventions, and pathologic diagnosis.

	Pregnant (n=12)	Non-pregnant (n=60)	p-Value
More than 1 ED visit	16.7 (2)	1.7 (1)	0.07
Seen initially as outpatient	8.3 (1)	28.3 (17)	0.27
Transferred from another hospital	25.0 (3)	0 (0)	<0.01
Pain medication given	83.3 (10)	71.7 (43)	0.50
More than 1 imaging study	75.0 (9)	15.0 (9)	<0.01
How diagnosis made			<0.01
CT	16.7 (2)	90 (54)	
MRI	33.3 (4)	0 (0)	
Ultrasound	33.3 (4)	3.3 (2)	
Clinical diagnosis	16.7 (2)	6.7 (4)	
Timing of interventions			
From presentation to first imaging study, hours	8.0 ± 11.1	3.2 ± 2.0	0.16
From presentation to antibiotics, hours	14.5 ± 12.0	5.9 ± 3.2	<0.01
From initial imaging study to operating room, hours	12.0 ± 8.9	6.7 ± 4.6	0.66
From presentation to operating room, hours	20.0 ± 11.8	9.9 ± 4.9	0.01
Length of surgery, minutes	54.8 ± 31.3	45.6 ± 19.5	0.34
Length of hospital stay (hours) – mean	65.9 ± 39.1	28.8 ± 15.9	<0.01
Length of hospital stay (hours) – median	67.2 (30.6, 84.7)	25 (18.4, 33.4)	<0.01
Length of postoperative stay (hours) – mean	51.1 ± 34.9	18.6 ± 14.3	<0.01
Length of postoperative stay (hours) – median	56.1 (22.2, 63.3)	16 (9.6, 21.2)	<0.01
Type of surgery			<0.01
Laparoscopic	58.3 (7)	100 (60)	
Laparoscopic converted to laparotomy	16.7 (2)	0 (0)	
Laparotomy	25.0 (3)	0 (0)	
Pathologic diagnosis			
Acute appendicitis	100 (12)	100 (60)	
Chronic appendicitis	0	5.0 (3)	1.0
Perforation	25.0 (3)	3.3 (2)	0.03

Data presented as % (n), mean + SD, and median and interquartile range, where applicable. ED, emergency department; CT, computerized tomography scan; MRI, magnetic resonance imaging. Three nonpregnant patients with chronic appendicitis had acute infection superimposed on chronic inflammation on pathology.

Table 4 shows the available pregnancy outcomes of the 12 patients. Five patients had a term vaginal delivery, and two patients had a term cesarean delivery. All seven neonates were appropriately grown for gestational age. Two patients underwent termination of pregnancy: one for trisomy 13 and the other for congenital cytomegalovirus infection. Three patients were delivered at an outside institution and delivery information could not be obtained.

**Table 4:** Pregnancy outcomes.

Case	Gestational age at diagnosis of appendicitis, weeks	Pregnancy outcome	Birth weight, grams
1	26	Vaginal delivery at 39 weeks	3,822
2	8	Termination for trisomy 13	
3	3	Termination for congenital CMV	
4	13	Delivered at outside institution	
5	28	Vaginal delivery at 37 weeks	3,120
6	31	Vaginal delivery at 40 weeks	3,742
7	11	Cesarean delivery at 39 weeks	3,335
8	30	Delivered at outside institution	
9	31	Repeat cesarean at 39 weeks	3,300
10	24	Vaginal delivery at 40 weeks	3,590
11	16	Vaginal delivery at 40 weeks	3,345
12	16	Delivered at outside institution	

## Discussion

Pregnant patients with appendicitis present in a similar fashion to nonpregnant patients, yet it took twice as long to achieve surgical management. Furthermore, pregnant patients had a higher perforation rate than nonpregnant patients. We cannot conclude that the delay in surgical management was associated with a higher perforation rate; however, the delay may have had some effect. Though presentations were quite variable, the groups did not differ, and only half of patients in each group presented with right lower quadrant pain. The majority in both groups had symptoms for only a day and nausea was the most common symptom. Both groups had similar leukocytosis and neutrophilia and only seven of 72 patients exhibited temperature >100.5 °F. The variability in the clinical presentations explains the strong reliance on imaging.

Previous studies have compared pregnant to nonpregnant patients with acute appendicitis [3], 9]. Abbasi et al., utilizing data from the Health Care Cost and Utilization Project, Nationwide Inpatient Sample, showed an almost two-fold increase in adverse outcomes such as sepsis, septic shock, transfusion, pneumonia, bowel obstruction, postoperative infection, and length of stay >3 days compared to nonpregnant patients [3]. Segev et al. retrospectively compared pregnant patients who underwent appendectomy for presumed acute appendicitis to a control group of nonpregnant patients [9]. There were no differences in WBC count, time from onset of symptoms to admission, admission to surgery, or operative time. More pregnant patients had neutrophilia and nonpregnant patients had higher mean temperature. US was used more often in pregnant patients and CT more often in nonpregnant patients. The authors concluded that pregnant and nonpregnant patients have similar perioperative management algorithms [9].

Preoperative imaging is an important component of making the diagnosis, but the modalities used in pregnancy, US, and MRI, do not have the same sensitivity as CT. The accuracy of CT for diagnosing appendicitis in adults with suspected appendicitis was evaluated by Rud et al. in a systematic review. CT had a summary sensitivity of 0.95 (95 % CI (Confidence Intervals) 0.93–0.96) and summary specificity of 0.94 (95 % CI 0.92–0.95) [10]. They reported that the probability of having appendicitis following a positive CT result was 0.92 (95 % CI 0.90–0.94) and the probability of having appendicitis after a negative CT result was 0.04 (95 % CI 0.03–0.05) [10]. In a systematic review and meta-analysis on the use of MRI for diagnosis of appendicitis in pregnancy, Kave et al. reported MRI had a sensitivity of 91.8 % and specificity of 97.9 % [11]. MRI has emerged as the first line imaging modality for appendicitis in pregnancy.

Access to MRI is not equivalent to CT at our institution. It took on average 5 h longer to obtain an MRI, contributing to the significant difference in time from presentation to surgery between groups. There were two pregnant patients diagnosed with appendicitis by CT, both imaged less than 3 h from presentation. This was a deviation from hospital protocol but provides insight into the streamlined workflow of obtaining a CT for suspected appendicitis in our institution and illustrates the need for imaging protocols to decrease the length of time from presentation to imaging, prioritizing pregnant patients on the MRI schedule.

Pregnant patients in the first trimester and nonpregnant patients typically present to the Emergency Department and are evaluated by the emergency medicine physician who obtains a surgical consult after imaging confirms the clinical diagnosis. Pregnant patients that develop abdominal pain later in pregnancy present to the Labor and Delivery unit, initially evaluated by obstetricians with surgical consultation obtained after eliminating obstetrical etiologies. For the obstetrician, obtaining imaging prior to or concurrent with surgical consultation may help to decrease the time from presentation to surgery.

In nonpregnant patients, the preferred management of acute appendicitis is laparoscopic appendectomy [12]. Nonoperative management has been chosen in pregnant patients that are not suitable for surgery or have an uncomplicated appendicitis [13]. Abbasi et al. showed that pregnant patients should not be managed nonoperatively because their risk of sepsis, septic shock, peritonitis, and venous thromboembolism was higher compared to surgical management [3]. Similarly, using data from the National Inpatient Sample between January 2003 and September 2015, investigators found that successful nonoperative management was associated with higher odds of amniotic infection and sepsis compared with immediate surgery; there were no differences in preterm delivery, preterm labor, or abortion [14]. Failed nonoperative management during pregnancy that required surgery was associated with higher odds of preterm delivery, preterm labor, or abortion compared with immediate operation [14].

The Society of American Gastrointestinal Endoscopic Surgeons and the World Society of Emergency Surgery recommend laparoscopic appendectomy for acute appendicitis in pregnancy [5], 15]. Laparoscopy offers benefits for pregnant patients including shorter hospital stay and similar maternal outcomes compared to laparotomy [5]. Yang et al. showed no increased risk of abortion with laparoscopy compared to laparotomy [13]. In our study, all nonpregnant patients underwent laparoscopic appendectomy. Nine of the pregnant patients underwent laparoscopy with two converted to laparotomy while the remaining three had laparotomy. Other studies share similar results [3], 9].

In our study, pregnant patients waited longer for surgical management, likely due to waiting longer for imaging, undergoing multiple studies and clinicians taking longer in deciding to proceed to surgery. In addition, pregnant patients had a longer postoperative stay and total hospitalization length of stay. Hiersch et al. reported a shorter interval between admission and surgery in pregnant patients compared to nonpregnant patients [16]. We did not show a difference in operative time between groups similar to other investigators [9]; while others have reported shorter surgical time in pregnant patients [16].

Future studies should look at whether more experience with the laparoscopic approach in pregnancy will increase the number of patients that undergo laparoscopy over laparotomy, whether imaging protocols will shorten the time from presentation to surgery in pregnancy and whether adverse fetal outcomes are related to complicated appendicitis vs. surgical approach.

A strength of this study is that it was done at a single institution with cases temporally matched to reflect similar management of patients by the same group of physicians. We were able to collect detailed information, particularly regarding time intervals for work up and treatment. This allowed objective assessment and recognition of the delay in treatment of pregnant patients. A limitation of the study is that since it was conducted at a single site it may not be generalizable. Review of the literature shows different imaging protocols and antibiotic protocols for appendicitis in pregnancy. Additionally, the small number of pregnant patients makes it difficult to comment on differences in presentation by trimester. Several pregnant patients did not deliver at our institution, so we have incomplete data on pregnancy outcomes.

Appendicitis should always be considered in any pregnant patient presenting with acute abdominal pain. Symptoms and physical examination in pregnant and nonpregnant patients are similar. There is a need for appropriate and prompt surgical treatment to avoid perforation with associated adverse outcomes, balanced with the need for additional imaging to enhance diagnostic accuracy to avoid morbidity associated with a negative appendectomy. Diagnostic delay significantly worsens prognosis, increasing the risk of maternal and fetal morbidity and mortality.
